# Which social determinants of health and long-term conditions contribute to inequalities in avoidable emergency hospital admissions? A decomposition analysis of small-area data in England

**DOI:** 10.1136/bmjph-2025-004560

**Published:** 2026-08-14

**Authors:** Morgan Beeson, Charlotte Parbery-Clark, Mark Lambert, John Wildman, Sarah Sowden

**Affiliations:** 1 NUBS, Newcastle University, Newcastle upon Tyne, UK; 2 Population Health Sciences Institute at Newcastle University, Newcastle University Faculty of Medical Sciences, Newcastle upon Tyne, UK; 3 NHS England, Newcastle upon Tyne, UK; 4 Newcastle University Business School, Newcastle upon Tyne, UK; 5 Population Health Sciences Institute, Newcastle University, Newcastle upon Tyne, UK

**Keywords:** Sociodemographic Factors, Emergencies, Demography

## Abstract

**Background:**

The burden of avoidable emergency admissions (AEAs) to hospital is an international concern. AEAs are unequally distributed across society with areas of socioeconomic disadvantage experiencing far more than their more affluent counterparts. Tackling inequalities in AEAs, through a better understanding of the contributing factors, could potentially free up resources for other parts of the health and care system. This study is the first to track income-inequality in AEAs over time and decompose which health and socio-economic factors contribute to those inequalities in England, an understanding of which is vital for policymakers to take effective action to reduce the burden of AEAs.

**Methods:**

Counts of AEAs for the 6,791 administrative areas in England for 2012/13 and 2018/19 were linked to household income, social determinants of health (SDOH) and the prevalence of long-term conditions (LTCs) data. Concentration indices were estimated to measure income-inequality in AEAs which were decomposed to estimate the contribution of income, age and sex, LTCs, SDOH factors and regional differences to income-inequality in AEAs.

**Results:**

The negative but increasing concentration indices indicate substantial but improving income-inequality in AEAs between 2012/13 and 2018/19. Over this time, income contributed to just a quarter of the observed income-inequality in AEAs. The aggregate contribution of SDOH factors rose from 39% to 51% while the contribution of the LTCs shrank from 20% to 7%. Significant, unexplained regional differences in AEAs indicate an ingrained North-South divide.

**Conclusion:**

Policy should target the reduction of AEAs in Northern post-industrial towns where the top contributing factors cluster.

**WHAT IS ALREADY KNOWN ON THIS TOPIC**

- Avoidable emergency admissions (AEAs) are costly and undesirable with significant and persistent inequalities which means there is great potential for cost saving and inequality reducing policy.

**WHAT THIS STUDY ADDS**

- Between 2012/13 and 2018/19, there was an 8% growth in AEAs in England.
- While remaining significant, income-inequality in AEAs shrunk by 10%; not due to improved conditions for the worse-off in society but through worsening conditions for the better-off in a ‘levelling down’ effect.
- Social determinants of health factors make the greatest contribution to income-inequality in AEAs which increased over the study period (39% to 51%), while the contribution of long-term conditions decreased (20% to 7%) and was overtaken by the contribution of unexplained regional differences (12% to 19%) suggesting social and geographic inequalities are becoming more entrenched.

**HOW THIS STUDY MIGHT AFFECT RESEARCH, PRACTICE OR POLICY**

- Interventions aimed at reducing inequality should target populations with clusters of the key contributing factors: low household income, low educational qualifications, many routine and technical jobs, high unemployment and high rates of chronic obstructive pulmonary disease.

## Introduction

 Due to the high associated costs, healthcare systems worldwide are making considerable efforts to reduce emergency admissions, many of which can be considered avoidable. Each year in the EU, 37 million bed days are used due to potentially avoidable admissions for chronic disease which diverts resources away from elective hospital care^1^ and care in the community. Avoidable emergency admissions (AEAs) are those for long-term conditions (LTCs) such as diabetes, dementia, epilepsy, angina and chronic obstructive pulmonary disease (COPD) and for avoidable accidents such as trips and falls.^2^ The list of disease codes used to define AEAs in England, which is the setting for this study, are provided in the online supplemental material table A1. AEAs should be amenable to public health, primary care, social care and emergency care interventions and therefore reflect the effectiveness of health and disease prevention and management.^2^ Targeting the reduction of AEAs would be a clear implementation of the recent 10-year plan for the NHS in England,^3^ which seeks shifts from hospital to community care and from a focus on sickness to prevention.

In England, AEAs are unequally distributed across society with people living in areas of socioeconomic disadvantage, measured using the Index of Multiple Deprivation (IMD), experiencing higher rates of AEAs than those living in areas of socioeconomic advantage.^4–6^ Inequalities are expensive with inequality in inpatient care in England costing £4.8 Billion a year in 2011/12.^7^ Intervening to address inequalities in health requires action on the wider socio-economic determinants of health (SDOH) including income, employment, education, housing and support networks.^8–11^ Qualitative evidence suggests that AEAs are no exception with a complex mix of primary and community care determinants as well as wider determinants, such as financial constraints to public services, residential gentrification and health and care expectations.^12,13^ The intervening years between 2010 and 2019 in England are often characterised by the austerity policies which cut local public health spending, social care and other local services. Services that generally promote the maintenance of health and well-being in the community. The impact of austerity varied widely by region, often hitting the least affluent areas the hardest; particularly the former industrial towns and cities of the North of England.^14^

While the prevalence of LTCs that contribute to AEAs are greater in socioeconomically disadvantaged areas,^15^ there is limited evidence to assess what degree of inequality in AEAs can be explained by inequality in LTCs compared with SDOH factors, and whether and how this may change over time. While the IMD is a useful composite measure encompassing many health and SDOH factors, identifying the separate SDOH factors that contribute to inequality in AEAs will help policymakers develop policies to intervene to improve health and reduce health inequalities in the community. To that end, this paper aims to measure income-inequality in AEAs between 2012/13 and 2018/19 and determine which LTCs and SDOH factors have contributed.

## Materials and methods

### Avoidable emergency admissions

Counts of AEAs between April 2012 and March 2013 and between April 2018 and March 2019 at lower layer super output area (LSOA) level are sourced from Hospital Episode Statistics (HES) Admitted Patient Care.^16^ AEAs are a subset of emergency admissions defined by the ICD-10 codes listed under Indicator 106a of the Clinical Commissioning Group Improvement and Assessment Framework (full list in the online supplemental material table A1). These ICD-10 codes are for chronic ambulatory care sensitive conditions and urgent care sensitive conditions which are overlapping groups including COPD, angina, heart failure, atrial fibrillation, asthma, epilepsy, abdominal and pelvic pain, urinary tract infections and acute mental health crises. Counts are aggregated from the LSOA level to the middle layer super output area (MSOA) level which are two levels of census area. MSOAs have populations of approximately 8000, roughly the size of towns or city quarters. The final sample includes the full population of 6791 MSOAs in England for both years.

### Measuring income-inequality in AEAs using concentration indices

Household income is used to measure income-inequality in AEAs. The Office for National Statistics (ONS) produces estimates of household income at the MSOA level using survey data from the Family Resources Survey and several national datasets.^17^ Income is logged to account for skewness.

Concentration indices are used to measure income inequality in AEAs. The Kakwani *et al*^18^ approach is used to estimate the concentration indices and the associated standard errors (SEs) (see the online supplemental material section B for details). Negative values (−1 to zero) indicate that AEAs are more concentrated among low-income MSOAs and positive values (zero to 1) indicate that AEAs are more concentrated among high-income MSOAs. Zero indicates equality. The hypothesis proposed in this study is that AEAs are more concentrated in low-income MSOAs and therefore a statistically significant negative concentration index is expected.

### Decomposition of income-inequality in AEAs

The proposed explanation for the above hypothesis is that the LTCs (that could result in an AEA) are more prevalent, more underdiagnosed and have poorer management in lower-income MSOAs. Further, SDOH factors, such as education, employment and living conditions, likely contribute to the relationship between income and the prevalence, diagnosis and management of LTCs.

To quantify the relative contributions of diagnosed LTCs and SDOH factors to income-inequality in AEAs, the Wagstaff *et al*^19^ approach is applied. This approach additively decomposes the concentration indices for AEAs (see the online supplementary material, Section C for details), calculating the percentage contribution of each LTC and SDOH factor to income-inequality in AEAs. The relative size of a factor’s contribution depends simultaneously on the strength of association with AEAs and the income-inequality of the factor. For example, if MSOAs with lower levels of education attainment generally have higher rates of AEAs and lower incomes then education will make a strong contribution to income-inequality in AEAs.

The income-inequality of each contributing factor is estimated using concentration indices as described in the previous section. The associations between each contributing factor and AEAs are estimated using a determinants of healthcare utilisation model with AEA counts as the dependent variable and the potential contributing factors as independent variables. The model is estimated using ordinary least squares with robust SEs. Combining the concentration indices and model coefficients from the determinants of care utilisation model using the equation in the online supplemental material section C gives the contribution of each factor to the concentration index of AEAs in percentage terms. A factor’s contribution is the proportion of the concentration index, which measures income-inequality in AEAs, that is accounted for by that factor.

The potential contributing factors are generated from data from the ONS, the Place Based Longitudinal Data Resource (PLDR) and the 2011 census. Details of the variables and their construction are in the online supplemental material section D table D1 and table D2. Population estimates of twelve age-sex groups for each MSOA are sourced from the ONS. The prevalence of LTCs is provided by the PLDR based on the GP Quality and Outcomes Framework reporting.^20–24^ Asthma, atrial fibrillation, COPD, coronary heart disease (CHD) and depression were chosen as these conditions contribute to roughly half of all AEAs (the associated ICD-10 codes are listed in bold font in the online supplemental material table A1). Prevalence data from the previous years (2011/12 and 2017/18) are used to estimate the effect of previously diagnosed LTCs because some diagnoses are made due to the AEA, making the AEA less preventable in the community. Data from the 2011 census are used to produce variables describing each MSOA’s SDOH in terms of the predominant social class, education level, broad ethnic group, as well as the unemployment rate, the proportion of single-person households and the rural-urban classification.^25^ The SDOH factors taken from the census are assumed to be time-invariant for the purpose of the study, as the next census collection was not until 2021. Regional indicators are also included, which will capture any unobserved, spatially clustered determinants not included in the model.

## Results

**Figure 1:**
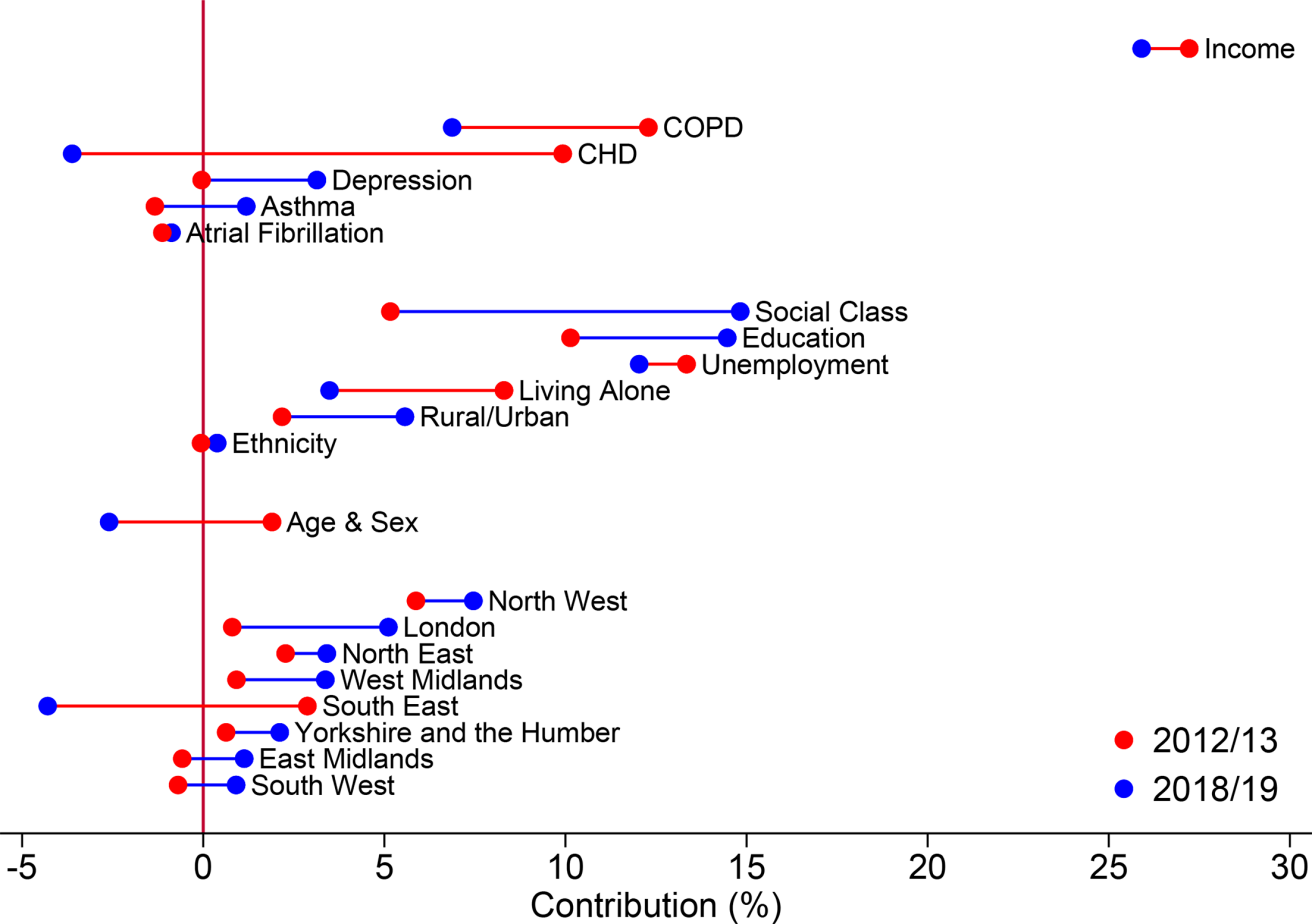
The percentage contribution of each factor to income inequality in avoidable emergency admissions in 2012/13 and 2018/19. CHD, coronary heart disease; COPD, chronic obstructive pulmonary disease.

**Figure 2:**
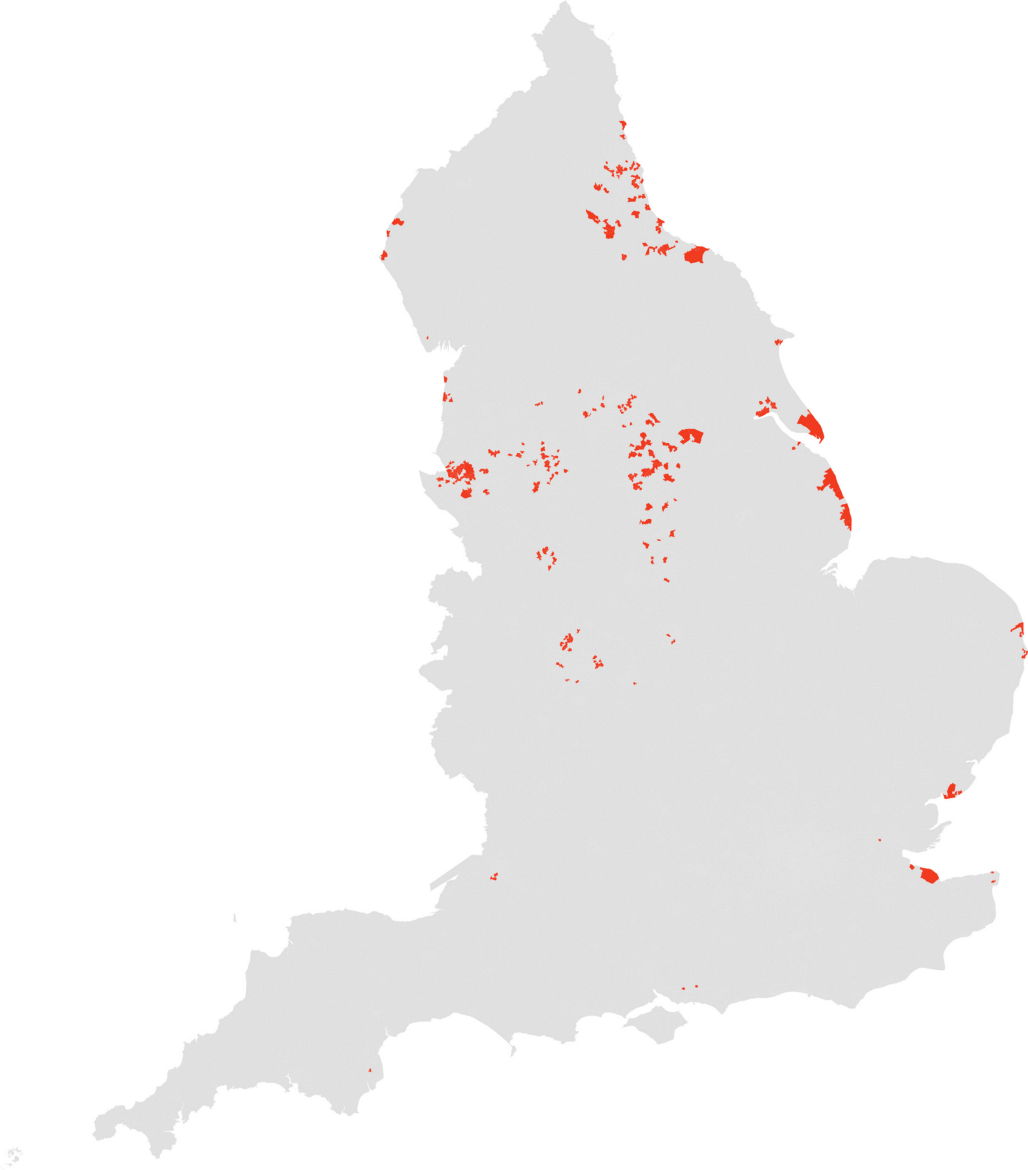
Middle layer super output areas with the top 5 social determinants of health and long-term condition contributing factors in 2018: low household income (bottom quintile), high unemployment (top quintile), high prevalence of chronic obstructive pulmonary disease (top quintile) and where no educational qualifications and manual and routine occupations (NSSEC3-3) are the most prevalent categories.

The mean count of AEAs, the concentration index for AEAs and the decomposition of income-inequality in AEAs into the broad contributions of income, LTCs, SDOH factors, age, sex and region are presented by year in table 1. The decomposition results are presented in greater detail in figure 1 which reports the contributions of individual factors and the change over time. The determinants of healthcare utilisation model and concentration indices used to calculate the percentage contributions are presented in the online supplemental material section E table E1 and table E2. Figure 2 is a map of the MSOAs that are experiencing the top contributing factors of inequality highlighted in red.

**Table 1:** Results: sample means, concentration indices and percentage contributions to income-inequality in avoidable emergency admissions for 2012/13 and 2018/19

Year	2012	2018
Mean (SD)	212.1 (82.2)	230.0 (90.7)
Concentration Index (SE)	−0.120 (0.002)	−0.108 (0.002)
Decomposition contribution		
Income	27%	26%
LTCs	20%	7%
SDOH	39%	51%
Age/sex	2%	−3%
Region	12%	19%
**Total**	**100%**	**100%**

LTCs, long-term conditions; SDOH, social determinants of health.

The concentration indices and associated SEs are estimated based on the online supplemental supplementary material,equation 2. All concentration indices are significant at the 99% confidence level. The percentage contributions are calculated as a proportion of the concentration index for AEAs based on the online supplemental material,section C, equation 4. The contribution of the LTCs (COPD, CHD, Depression, Asthma, Atrial Fib.), SDOH factors (Ethnicity, Education, Social Class, Unemployment, Lives Alone, Urban) and regions (East of England as reference) are reported as aggregates in bold.

AEAs are prevalent with a mean count per MSOA of 212 in 2012/13. This amounts to 269 per 10 000 and accounts for 29% of all emergency admissions. The concentration index of −0.12 (p<0.001) indicates there is income-inequality in AEAs. The negative concentration index means that AEAs are more concentrated in lower-income MSOAs. While between 2012/13 and 2018/19, the number of AEAs increased by 8% to 230 per MSOA, income-inequality in AEAs reduced by 10% to a concentration index of 0.108 (p<0.001). The reduction in inequality was due to worsening rates of AEAs in higher-income MSOAs relative to lower-income MSOAs in a ‘levelling-down’ effect.

The decomposition of these concentration indices provides some context to these changes. The contribution of income itself to income-inequality in AEAs was just 27% in 2012/13 and 26% in 2018/19. The remaining 74% (in 2018/19) of income-inequality in AEAs was accounted for by LTCs, SDOH factors, age and sex and regional differences. These factors contribute because they are simultaneously strong determinants of AEAs and exhibit income inequalities.

Despite the LTCs included in the analysis making up half of AEAs (see the online supplemental material table A1), the contribution of previously diagnosed LTCs to income inequality in AEAs is relatively small at 20% in 2012 and reducing to 7% by 2018. Figure 1 shows that COPD and CHD made large but reducing contributions while the contribution of depression increased, indicating a shift from physical health to mental health contributing to income inequality in AEAs. The reduced contribution of LTCs is displaced by increased contributions of SDOH factors (39% to 51%) and unexplained regional differences (12% to 19%). Social class, education and unemployment make the greatest contributions of the SDOH factors. The detailed results in the online supplemental material section E show that the contribution of social class is almost entirely due to the lower income and higher rates of AEAs in MSOAs where routine and manual occupations are most common (NSSEC-3 Level 3), which makes up 55% of MSOAs. The contribution of education is due to MSOAs where it is most common to be without educational qualifications, which makes up 15% of MSOAs. Widening regional differences in income and AEAs (after controlling for LTCs and SDOH factors) over the study period produced the increased contribution of region. The contribution of age and sex remains small throughout the study period because, while MSOAs with older populations tend to have more AEAs, they also tend to have higher incomes and therefore age and sex do not contribute to income-inequality in AEAs.

The MSOAs that fare the worst in terms of inequality of AEAs are those with low household incomes where rates of COPD are high, educational attainment is low, jobs are mostly routine and manual occupations and there is high unemployment. These factors contributed a combined 62% to income-inequality in AEAs in 2018. There are 364 MSOAs that have all these characteristics which is 5% of all MSOAs. These MSOAs, highlighted in red on the map presented in figure 2, are almost all located in urban centres in the North and Midlands that share industrial heritage and post-industrial economic challenges.

As a sensitivity analysis (see the online supplemental material section F tables F1-F7), the analysis is repeated for all-cause emergency admissions and the two subsets of AEAs: chronic ambulatory care sensitive conditions and urgent care sensitive conditions. Results show the same general pattern, but AEAs are more unequally distributed than other emergency admissions and chronic ambulatory care sensitive conditions display particularly severe income-inequality.

As the AEA variable is count data, as a sensitivity analysis, the determinants of healthcare utilisation models are estimated using a negative binomial model. The marginal effects estimates are then used in place of the coefficients from the main model to calculate the percentage contributions. The estimates are presented in Section G of the online supplemental material table G1 and figure G1 presents the contributions, which when compared with figure 1, show very similar results.

## Discussion

### Summary of results

This paper studies AEAs which are a prevalent subset of emergency admissions with significant inequalities. Our results show that there were 1.56 million AEAs in 2018/19. At an average cost of £3,293 per non-elective inpatient,^26^ this amounts to £5.14 billion or £106 per person over 16 years old. The concentration indices, which measure income-inequality in AEAs at the MSOA-level, were negative indicating AEAs are more concentrated in lower-income MSOAs. If the rate of AEAs were the same for lower-income MSOAs as those MSOAs in the top quintile of income, there would have been 380 thousand fewer AEAs saving £1.24 billion in 2018/19.

We identified worsening rates of AEAs but improving income-inequality in AEAs between 2012/13 and 2018/19. Over that period AEAs did worsen across the income distribution at an average of 8% but the growth rate was faster in higher-income MSOAs. This produced a pattern consistent with a ‘levelling-down’ effect, whereby inequality improved because AEAs increased more rapidly in higher-income MSOAs rather than due to fewer AEAs in lower-income MSOAs. However, alternative explanations may also contribute to this pattern, including changes in clinical admission thresholds, NHS system pressures, and changing healthcare access and utilisation.

The main contribution of this paper is the decomposition of income-inequality in AEAs into contributions of different factors. In 2018, the top five contributing SDOH and LTC factors were household income, social class, educational attainment, unemployment and the prevalence of COPD. Contributing factors are those associated with both higher AEAs and lower household incomes. These factors cluster together in post-industrial towns and cities in the Midlands and North of England. The region effects identified that living in the North West was a particularly strong contributing factor to inequalities, likely reflecting broader unobserved and spatially clustered structural and contextual differences between northern and southern regions of England. These were the areas most affected by austerity policy.^14^ This result is part of a larger pattern indicting a North-South divide where, after controlling for differences in SDOH factors and LTC prevalence, northern areas had more AEAs and lower incomes than their southern counterparts.

The contribution of diagnosed LTCs reduced although the contribution of depression did increase. This could indicate that LTCs are generally being better managed in the community resulting in a reduced contribution to income-inequality in AEAs. Alternatively, there may be under-diagnosis or relatively worse management of LTCs in MSOAs where educational attainment is low and employment prospects are poor.

### Comparison to other literature

The concentration indices reported in this study are relatively large in absolute terms compared with other studies measuring income-inequality in health outcomes, for example, 0.04–0.07 for self-assessed health in England between 1997–2007, indicating that inequality in AEAs is particularly substantial.^27^ Other studies of inequality in AEAs showed stable IMD-based inequality in AEAs between the English Local Authorities (LAs, local government regions) from 2004 to 2011,^4^and improving inequality between 2010 and 2017.^5^ These studies differ as they track within-LA inequality measured by IMD, a composite measure of socio-economic status. The latter study overlaps in time period with the current study, also observing a ‘levelling-down’ effect where the least deprived LAs see relative increases in AEAs.^28^ Moving beyond the current study period, the COVID-19 pandemic was found to reinforce inequalities.^6^

The results from the decomposition add to the growing evidence of the dual geographic inequalities in wealth and health in England, which culminated in stark differences in the experience of the COVID-19 pandemic between the North and South.^29^ Recent studies have shown people believe this North-South divide to be deeply rooted social issues around deindustrialisation, unemployment, loss of community facilities and disengagement from politics.^30,31^ The hypothesis of under-diagnosis and/or relatively worse management of LTCs in socio-economically disadvantaged communities is supported by reports of patients with poor mental health, living in areas of extreme socioeconomic disadvantage deprioritising health due to social issues in their lives leading to poorer management of their conditions.^32,33^ The increased contribution of depression to inequalities identified in this study aligns with increases in deaths of despair over the same time period,^34^ which share similar determinants such as unemployment and routine and manual occupations,^35^ and have been linked to austerity policy.^36^

## Limitations

This study was an area-level analysis that estimates associations between healthcare utilisation and the population’s health and SDOH with secondary data, some of which was either self-reported or estimated and not necessarily generated for the purpose of this study. The AEA measure used in this study is a standard NHS England indicator used for healthcare quality and inequality monitoring. However, the term “avoidable” should not be interpreted as implying that every individual admission is preventable. Rather, AEAs are more appropriately interpreted as a population-level indicator of potentially preventable or system-sensitive emergency admissions. Particularly in ageing populations with increasing multimorbidity, some exacerbations and emergency admissions, such as one following a fall, may be clinically unavoidable despite optimal care. Care must be taken when interpreting these results to avoid assuming causality, the ecological fallacy, and understanding that errors may have been introduced during data collection. Individual survey data in England does not include AEAs and the routinely collected HES data does not include many of the SDOH variables of interest, such as income, education and household characteristics. Instead, SDOH variables were derived from the 2011 Census and therefore treated as time-invariant across the study period. These variables should therefore be interpreted as representing relatively stable structural characteristics of areas rather than annually varying exposures. While these characteristics change gradually over time, there may be some change, and this should be considered when interpreting changes in decomposition contributions between 2012/13 and 2018/19. Furthermore, while the study incorporated several important SDOH measures, there may be other potentially relevant ethnocultural and social dimensions such as refugee status and migration experience that may contribute to inequalities and warrant further investigation in future research. Despite these limitations, area-level data is useful for describing the conditions in a community. While establishing causality would be preferable, health inequality by income, education, occupation, etc. may be regarded as unfair regardless of whether there is a causal relationship established. While the study period covered an interesting period of reduced government spending, it is now 6 years from the end of the study period. As more recent studies have found worsening inequality during the COVID-19 pandemic,^6^ the estimated inequality in this study may be an underestimate of the current levels.

### Policy and research implications

Policy to reduce AEAs could simultaneously improve inequality and be cost saving. In the short term, policy should target the towns and cities in the Midlands and North of England where there are clusters of low educational attainment, high unemployment, poorly paid jobs and high rates of COPD. Given the opportunity to intervene in the community before AEAs occur, these locations would make good potential sites for the neighbourhood health services set out in the recent 10 year plan for the NHS which strives for fairer geographic distribution of funding.^3^ Future research should explore the potential causal relationships between these factors and AEAs. In the long term, policies that provide equality of opportunity for education and employment could reduce these inequalities substantially.

## Conclusion

There were over 1.5 million AEAs in England costing over £5 billion in 2018. AEAs are unequally distributed across society with individuals of lower socio-economic status more likely to have an AEA. Despite the worsening rates of AEAs during the years of austerity in England, there was an improvement in income-inequality in AEAs due to a “levelling-down” effect. Through the decomposition of these inequalities, we have shown that the contributions of the prevalence of diagnosed LTCs are declining while the contributions of SDOH factors are increasing. The top five contributing factors were household income, social class, education, unemployment and COPD. There is also evidence that the North-South divide is growing. Short-term policy should target the post-industrial towns and cities in the Midlands and North of England where the greatest contributing factors are clustered. Reducing AEAs in these areas could be simultaneously inequality improving and cost-saving. We provide additional evidence that longer-term policies should seek to improve the equality of opportunity for education and employment.

## Supplementary material

10.1136/bmjph-2025-004560 online supplemental file 1

## Data Availability

Data may be obtained from a third party and are not publicly available.
