# Supplementary material for: Which social determinants of health and long-term conditions contribute to inequalities in avoidable emergency hospital admissions? A decomposition analysis of small-area data in England
Source: BMJ Public Health. 2026 Aug 14;4(3):e004560. doi: 10.1136/bmjph-2025-004560 (PMC13479504; doi:10.1136/bmjph-2025-004560)
Supplement: Supplementary file 1 [file bmjph-4-3-s001.pdf]

Which social determinants of health and long-term conditions contribute to inequalities in avoidable emergency hospital admissions?

Supplementary Material

A. AEAs definition
B. Concentration Indices
C. Decomposition approach
D. Data and variables
E. Full results
F. Sensitivity analysis 1 – Alternate emergency admission measures.
G. Sensitivity analysis 2 – Using a negative binomial model to estimate the determinants of health care utilisation model.

A. AEAs definition

Table A1. ICD-10 codes for AEAs with % of total AEAs for 2012/13 and 2018/19.

ICD-10	Name	12/13	18/19
B18.0	Chronic viral hepatitis B with delta-agent	<0.00%	<0.00%
B18.1	Chronic viral hepatitis B without delta-agent	<0.00%	0.01%
D50.1	Sideropenic dysphagia	<0.00%	0.00%
D50.8	Other iron deficiency anaemias	0.37%	0.42%
D50.9	Iron deficiency anaemia, unspecified	0.56%	1.08%
D51	Vitamin B12 deficiency anaemia	0.02%	0.02%
D52	Folate deficiency anaemia	0.05%	0.07%
E10	Insulin-dependent diabetes mellitus	1.63%	1.71%
E11	Non-insulin-dependent diabetes mellitus	1.17%	1.47%
E12	Malnutrition-related diabetes mellitus	<0.00%	<0.00%
E13	Other specified diabetes mellitus	0.02%	0.03%
E14	Unspecified diabetes mellitus	0.06%	0.06%
E15	Nondiabetic hypoglycaemic coma	<0.00%	0.00%
E16.1	Other hypoglycaemia	0.02%	0.04%
E16.2	Hypoglycaemia, unspecified	0.86%	0.84%
F00	Dementia in Alzheimer disease	0.09%	0.07%
F01	Vascular dementia	0.28%	0.20%
F02	Dementia in other diseases classified elsewhere	0.01%	0.01%
F03	Unspecified dementia	0.38%	0.31%
**F04-99**	**Acute mental health crisis**	**9.09%**	**9.04%**
G40	Epilepsy	2.49%	2.18%
G41	Status epilepticus	0.24%	0.25%
**I10X**	**Essential (primary) hypertension**	**0.55%**	**1.29%**
**I11.0**	**Hypertensive heart disease with (congestive) heart failure**	**0.02%**	**0.02%**
**I11.9**	**Hypertensive heart disease without (congestive) heart failure**	**0.01%**	**0.01%**
**I13.0**	**Hypertensive heart and renal disease with (congestive) heart failure**	**<0.00%**	**0.01%**
**I20**	**Angina pectoris**	**4.24%**	**2.60%**
**I25**	**Chronic ischaemic heart disease**	**1.09%**	**0.80%**
**I48**	**Atrial fibrillation and flutter**	**4.31%**	**4.46%**
**I50**	**Heart failure**	**4.17%**	**5.00%**
I80-I82	Deep vein thrombosis	1.47%	1.80%
J20	Acute bronchitis	0.19%	0.23%
J40	Bronchitis, not specified as acute or chronic	0.14%	0.14%
J41	Simple and mucopurulent chronic bronchitis	<0.00%	<0.00%
J42	Unspecified chronic bronchitis	0.01%	0.01%
J43	Emphysema	0.17%	0.16%
**J44**	**Other chronic obstructive pulmonary disease**	**8.31%**	**8.48%**
**J45**	**Asthma**	**4.20%**	**4.12%**
**J46X**	**Status asthmaticus**	**0.41%**	**0.32%**
J47X	Bronchiectasis	0.50%	0.62%
J81X	Pulmonary oedema	0.13%	0.08%
L03	Cellulitis	4.37%	5.92%
N39.0	Urinary tract infection, site not specified	11.14%	10.48%
R07.2	Precordial pain	2.82%	3.34%
**R07.3**	**Other chest pain**	**4.92%**	**4.89%**
**R07.4**	**Chest pain, unspecified**	**9.71%**	**8.37%**
R10	Abdominal and pelvic pain	16.34%	16.29%
R50*	Pyrexial (Patients aged 6 years or under)	-	-
S00	Superficial injury of head	2.79%	2.75%
T80	Blocked tubes, catheters and feeding tubes	0.60%	0.04%
W0-19*	Falls (Patients aged 74 years or over)	-	-

Notes: From https://digital.nhs.uk/data-and-information/publications/statistical/hospital-admitted-patient-care-activity. *No data was available for age specific categories. Bold codes are for diseases related to COPD, CHD, depression, asthma, and atrial fibrillation.

B. Concentration Indices

For empirical estimation, the concentration index, CI, is defined as twice the covariance between the variable of interest, x, and rank of income divided by the mean of the x as shown by

[1] $$CI_x = \frac{2}{\mu} cov(x, R)$$

Where μ is the sample mean of x across the sample and R is the income rank. CI_x takes a value between -1 and 1, where negative values indicate that admissions are more concentrated among low income areas and a value of 0 designates equality.

Following, Kakwani et al.[1], the concentration index and standard error are estimated using ordinary least squares (OLS) run on the following equation:

[2] $$2\sigma_R^2 \left[\frac{x_{msoa}}{\mu}\right] = \alpha + \gamma R_{msoa} + u_{msoa}$$

Where σ_R^2 is the variance of relative rank, α is the intercept term, and R_{msoa} is the MSOA's relative income rank.

The Wagstaff approach[2] is used when the variable of interest, x, is binary. The **conindex** command in Stata18 is used to estimate concentration indices.

[1]Kakwani N, Wagstaff A, Van Doorslaer E. Socioeconomic inequalities in health: measurement, computation, and statistical inference. Journal of econometrics. 1997 Mar 1;77(1):87-103.

[2] Wagstaff A. The bounds of the concentration index when the variable of interest is binary, with an application to immunization inequality. Health economics. 2005 Apr;14(4):429-32.

C. Decomposition approach

The decomposition approach[3] requires concentration indices for each potentially contributing factor and the coefficients from a determinants of health care utilisation model. The determinants of health care utilisation model estimate the associations between an area's population and geographic characteristics and admissions and follows the equation:

[3] $$AEA_{msoa} = \beta_0 + \beta' X_{msoa} + \varepsilon_{msoa}$$

Where AEA_{msoa} is the count of admissions in each MSOA, β is a vector of l coefficients which represent the partial effects of exogenous regressors X_{msoa}, β_0 is the intercept, and ε_{msoa} is the error term. The exogenous regressors include population counts by age and sex, socio-economic status, the prevalence of specific long-term conditions. Region dummies capture some of the remaining geographic variation in admissions that cannot be explained by variation in income, socio-economic status, and disease prevalence. The model is estimated using OLS with robust standard errors.

The decomposition can be written as

[4] $$C_H = \sum_{l=1}^{T} \left(\frac{\beta_l \bar{X}_l}{\mu}\right) C_{X_l} + \left(\frac{\bar{\varepsilon}}{\mu}\right) C_\varepsilon$$

Where C_H is the concentration index of AEAs. Concentration indices, C_{X_l}, are estimated for each explanatory variable. β_l comes from a determinants of health care utilisation model defined in Equation 3 above. $\mu, \bar{X}_l, \bar{\varepsilon}$ are the sample means of the count of AEAs, the lth independent variable, and the residual respectively. The contribution of an individual determinant is $\left(\frac{\beta_l \bar{X}_l}{\mu}\right) C_{X_l}$ which can then be calculated as a percentage of C_H.

[3] Wagstaff A, Van Doorslaer E, Watanabe N. On decomposing the causes of health sector inequalities with an application to malnutrition inequalities in Vietnam. Journal of econometrics. 2003 Jan 1;112(1):207-23.

D. Data and variables

The data sources are set out in Table D1. Data from Hospital Episode Statistics (HES) Admitted Patient Care, ONS population statistics, the Place Based Longitudinal Data Resource (PLDR), and the 2011 census provide AEA counts, income, age and sex, LTCs and SDOH factors.

Table D1. Data sources

Data	Source	Variables
AEAs	HES APC for 2012/13 and 2018/19	AEAs
Income	ONS estimates for 2011/12 and 2017/18[4]	Income (salaries, investment earnings, tax credits, pension, benefits, and other sources of income)
Population estimates	ONS estimates for 2012/13 and 2018/19[5]	Male0to17, Male18to34, Male35to49, Male50to64, Male65to79, Male80plus, Female0to17, Female18to34, Female35to49, Female50to64, Female65to79, Female80plus
SDOH / Region	2011 Census[6]	White (British), White (other), Black, Asian, Mixed/Other, Education Level 4, Education Level 3, Education Level ½, Education None, NSSEC3 Level 1, NSSEC3 Level 2, NSSEC3 Level 3, NSSEC3 Level 4, Unemployment, LivingAlone, Urban, Region
LTCs	Place-based longitudinal data resource. Estimates for 2011/12 and 2017/18[7]	Asthma, AtrialFibrillation, CHD, COPD, Depression

Table D2 describes the variables used in the analysis. AEAs are counts of admissions between April 2012 and March 2013 and between April 2018 and March 2019. There are population counts for 12 age-sex groups. The PLDR reports the prevalence of asthma, atrial fibrillation, COPD, CHD, and depression at the LSOA-level by attributing GP-level prevalence rates used for Quality and Outcomes Framework (QOF) reporting to the patients' LSOAs. Prevalence data is aggregated to the MSOA level accounting for different LSOA population sizes. As diagnoses of LTCs may occur at the time of the emergency admission, prevalence data from the previous year (2011/12 and 2017/18) so estimates are for previously diagnosed LTCs. Data from the 2011 census is used to produce variables describing MSOAs based on SDOH. 'Social class' is a categorical variable measuring the most prevalent classification of employment based on the NSSEC-3: Higher managerial, administrative and professional occupations (reference case); Intermediate occupations; Routine and manual occupations; or Never worked and long-term unemployed. 'Education' is a categorical variable measuring the most prevalent level of qualification: degree level or equivalent (reference case); A-levels and other post-16 qualifications; GCSEs; or no qualifications. 'Ethnicity' is a categorical variable measuring the most prevalent ethnic group from: White British (reference case); White other; Black; Asian; or mixed and other. 'Unemployment' is the proportion of 16 to 70-year-olds that are unemployed and actively seeking work which excludes those who are retired, long-term sick, looking after the home or family, and some full-time students. 'Living Alone' is the proportion of households that are single person households. 'Urban' is a dummy variable using the Rural-Urban Classification (RUC). Region is a categorical variable which is turned in a binary variable for each region.

[4]https://www.ons.gov.uk/employmentandlabourmarket/peopleinwork/earningsandworkinghours/datasets/smallareaincomeestimatesformiddlelayersuperoutputareasenglandandwales
[5]https://www.ons.gov.uk/peoplepopulationandcommunity/populationandmigration/populationestimates/datasets/lowersuperoutputareamidyearpopulationestimates
[6] https://infuse.ukdataservice.ac.uk/
[7] https://pldr.org/

Table D2. Variable descriptions

Variable	Description
AEAs	Count of avoidable emergency admissions
Income	Household income (logged)
Male0to17	Count of males aged between 0 and 17
Male18to34	Count of males aged between 18 and 34
Male35to49	Count of males aged between 35 and 49
Male50to64	Count of males aged between 50 and 64
Male65to79	Count of males aged between 65 and 79
Male80plus	Count of males aged 80 and over
Female0to17	Count of females aged between 0 and 17
Female18to34	Count of females aged between 18 and 34
Female35to49	Count of females aged between 35 and 49
Female50to64	Count of females aged between 50 and 64
Female65to79	Count of females aged between 65 and 79
Female80plus	Count of females aged 80 and over
Asthma	Prevalence (%) of asthma (QOF reporting)
AtrialFibrillation	Prevalence (%) of atrial fibrillation (QOF reporting)
CHD	Prevalence (%) of chronic heart disease (QOF reporting)
COPD	Prevalence (%) of chronic obstructive pulmonary disease (QOF reporting)
Depression	Prevalence (%) of depression (QOF reporting)
White (British)	White (British) is most prevalent ethnicity (categorical variable)
White (other)	White (other) is most prevalent ethnicity (categorical variable)
Black	Black (combined) is most prevalent ethnicity (categorical variable)
Asian	Asian (combined) is most prevalent ethnicity (categorical variable)
Mixed/Other	Mixed and other (combined) is most prevalent ethnicity (categorical variable)
Education Level 4	Qualification level 4 is most prevalent (categorical variable)
Education Level 3	Qualification level 3 is most prevalent (categorical variable)
Education Level 1/2	Qualification level 1 and 2 (combined) is most prevalent (categorical variable)
Education None	No qualifications is most prevalent (categorical variable)
NSSEC3 Level 1	NSSEC 3 category – category 1 is most prevalent (categorical variable)
NSSEC3 Level 2	NSSEC 3 category – category 2 is most prevalent (categorical variable)
NSSEC3 Level 3	NSSEC 3 category – category 3 is most prevalent (categorical variable)
NSSEC3 Level 4	NSSEC 3 category – category 4 is most prevalent (categorical variable)
Unemployment	Proportion of 16-70 year olds active in the labour market who are unemployed
LivingAlone	Proportion of households with only one person
Urban	Based on the rural urban classification (dummy variable)
EM	East Midlands (dummy variable)
EE	East of England (dummy variable)
LDN	London (dummy variable)
NE	North East (dummy variable)
NW	North West (dummy variable)
SE	South East (dummy variable)
SW	South West (dummy variable)
WM	West Midlands (dummy variable)
YRK	Yorkshire and The Humber (dummy variable)

E. Full results

Table E1. Expanded results for AEAs in 2012

Variable	Mean	s.d.	Beta	s.e.	Con. index	s.e.	Cont.	%
Logged Income	10.52	0.25	-52.65	7.87	0.014	0.000	-0.035	27%
Male 0 to 17	861.46	264.34	0.04	0.01	-0.012	0.002	-0.002	2%
Male 18 to 34	905.04	511.09	-0.01	0.00	-0.035	0.004	0.001	-1%
Male 35 to 49	816.84	211.10	0.05	0.01	0.028	0.002	0.005	-4%
Male 50 to 64	697.04	172.54	0.01	0.02	0.018	0.002	0.001	-1%
Male 65 to 79	457.57	168.94	-0.08	0.02	0.012	0.002	-0.002	2%
Male 80 plus	139.11	61.86	-0.03	0.04	0.016	0.003	0.000	0%
Female 0 to 17	820.41	252.83	0.03	0.01	-0.013	0.002	-0.001	1%
Female 18 to 34	896.58	473.44	0.02	0.01	-0.039	0.004	-0.003	3%
Female 35 to 49	830.34	201.18	-0.01	0.01	0.035	0.002	-0.001	1%
Female 50 to 64	714.86	183.19	0.02	0.02	0.022	0.002	0.002	-1%
Female 65 to 79	506.48	183.13	0.10	0.02	0.008	0.002	0.002	-1%
Female 80 plus	230.10	105.20	0.26	0.02	-0.010	0.003	-0.003	2%
COPD	1.65	0.61	15.73	2.07	-0.130	0.002	-0.016	12%
CHD	3.46	0.98	10.94	1.81	-0.072	0.002	-0.013	10%
Depression	5.84	1.86	-0.05	0.37	-0.041	0.002	0.000	0%
Asthma	5.98	0.85	-2.42	1.08	-0.025	0.001	0.002	-1%
Atrial Fibrillation	1.47	0.48	-20.14	3.06	-0.011	0.002	0.001	-1%
White (British)	Ref							
White (other)	0.00	0.07	-22.45	7.54	0.390	0.106	0.000	0%
Black	0.01	0.11	-26.68	7.86	-0.180	0.046	0.000	0%
Asian	0.04	0.21	-0.20	5.06	-0.152	0.037	0.000	0%
Mixed/Other	0.00	0.02	18.90	37.53	-0.630	0.394	0.000	0%
Education Level 4	Ref							
Education Level 3	0.03	0.17	12.98	6.31	-0.351	0.042	-0.001	1%
Education Level 1/2	0.45	0.50	2.27	1.99	-0.211	0.013	-0.001	1%
Education None	0.14	0.35	20.05	3.38	-0.853	0.020	-0.012	9%
NSSEC3 Level 1	Ref							
NSSEC3 Level 2	0.01	0.10	-6.47	5.41	0.165	0.054	0.000	0%
NSSEC3 Level 3	0.55	0.50	2.96	2.15	-0.851	0.006	-0.007	5%
NSSEC3 Level 4	0.00	0.03	35.75	16.66	-0.855	0.349	0.000	0%
Unemployment	0.06	0.03	275.88	54.67	-0.209	0.003	-0.017	13%
Living Alone	0.30	0.06	143.85	18.88	-0.053	0.002	-0.011	8%
Urban	0.82	0.38	2.67	1.90	-0.273	0.016	-0.003	2%
EoE	Ref							
SE	0.16	0.37	-11.03	2.23	0.440	0.018	-0.004	3%
NW	0.14	0.34	29.54	2.94	-0.403	0.020	-0.008	6%
Ldn	0.14	0.35	-2.93	2.91	0.521	0.019	-0.001	1%
WM	0.11	0.31	6.38	2.69	-0.366	0.021	-0.001	1%
NE	0.05	0.22	23.87	3.95	-0.524	0.036	-0.003	2%
EM	0.08	0.28	-5.96	2.80	-0.317	0.023	0.001	-1%
Yrks	0.10	0.30	7.30	2.66	-0.234	0.024	-0.001	1%
SW	0.10	0.30	-14.47	3.19	-0.128	0.019	0.001	-1%
Constant			474.44	90.31				

The concentration indices and associated standard errors (s.e.) are estimated based on Appendix B, Equation 2. The model coefficient (Beta) and associated standard errors (se) are estimated based on Appendix B, Equation 3. The absolute contributions (abs.) to income related inequalities in AEAs are calculated using Appendix B, Equation (4) and the percentage contributions are calculated as a proportion of the concentration index for AEAs.

Table E2. Expanded results for AEAs in 2018

Variable	Mean	s.d.	Beta	s.e.	Con. index	s.e.	Cont.	%
Logged Income	10.67	0.22	-62.25	10.20	0.012	0.000	-0.034	26%
Male 0 to 17	902.13	303.22	0.03	0.01	-0.004	0.002	-0.001	0%
Male 18 to 34	926.64	608.08	0.00	0.00	-0.027	0.005	0.000	0%
Male 35 to 49	796.95	265.19	0.02	0.01	0.046	0.002	0.004	-3%
Male 50 to 64	763.95	181.06	0.06	0.02	0.018	0.002	0.003	-3%
Male 65 to 79	520.20	193.67	-0.06	0.02	0.005	0.002	-0.001	1%
Male 80 plus	164.34	72.72	0.00	0.03	0.025	0.003	0.000	0%
Female 0 to 17	858.23	289.51	0.06	0.01	-0.004	0.002	-0.001	1%
Female 18 to 34	899.72	547.22	0.01	0.01	-0.031	0.005	-0.002	1%
Female 35 to 49	809.20	232.84	-0.03	0.02	0.046	0.002	-0.005	4%
Female 50 to 64	787.10	190.10	0.04	0.02	0.018	0.002	0.002	-2%
Female 65 to 79	571.03	209.73	0.04	0.02	0.009	0.002	0.001	-1%
Female 80 plus	243.36	108.31	0.32	0.02	0.004	0.003	0.001	-1%
COPD	1.93	0.71	7.61	2.39	-0.140	0.002	-0.009	7%
CHD	3.24	0.94	-4.15	2.28	-0.080	0.002	0.005	-4%
Depression	9.24	2.41	1.74	0.39	-0.058	0.002	-0.004	3%
Asthma	6.02	0.93	1.81	1.38	-0.033	0.001	-0.002	1%
Atrial Fibrillation	1.90	0.66	-6.47	2.89	-0.021	0.002	0.001	-1%
White (British)	Ref							
White (other)	0.00	0.07	-12.61	7.85	0.507	0.108	0.000	0%
Black	0.01	0.11	-37.26	8.14	0.063	0.044	0.000	0%
Asian	0.04	0.21	31.26	6.54	-0.068	0.038	0.000	0%
Mixed/Other	0.00	0.02	-197.63	58.89	-0.414	0.334	0.000	0%
Education Level 4	Ref							
Education Level 3	0.03	0.17	14.33	6.06	-0.356	0.042	-0.001	1%
Education Level 1/2	0.45	0.50	7.61	2.27	-0.226	0.014	-0.003	3%
Education None	0.14	0.35	27.14	4.16	-0.878	0.021	-0.015	11%
NSSEC3 Level 1	Ref							
NSSEC3 Level 2	0.01	0.10	5.39	5.79	0.098	0.062	0.000	0%
NSSEC3 Level 3	0.55	0.50	9.56	2.50	-0.832	0.006	-0.019	15%
NSSEC3 Level 4	0.00	0.03	102.09	14.48	-0.778	0.318	0.000	0%
Unemployment	0.06	0.03	288.83	64.99	-0.196	0.003	-0.016	12%
Living Alone	0.30	0.06	73.11	21.35	-0.048	0.002	-0.005	4%
Urban	0.82	0.38	12.09	2.11	-0.167	0.016	-0.007	6%
EoE	Ref							
SE	0.16	0.37	17.57	3.06	0.448	0.019	0.006	-4%
NW	0.14	0.34	44.51	3.41	-0.369	0.019	-0.010	8%
Ldn	0.14	0.35	-16.26	4.27	0.650	0.019	-0.007	5%
WM	0.11	0.31	31.92	3.89	-0.292	0.021	-0.004	3%
NE	0.05	0.22	35.03	4.54	-0.582	0.037	-0.004	3%
EM	0.08	0.28	20.94	3.36	-0.192	0.025	-0.001	1%
Yrks	0.10	0.30	17.33	4.09	-0.358	0.022	-0.003	2%
SW	0.10	0.30	20.35	3.15	-0.130	0.018	-0.001	1%
Constant			575.92	115.91				

The concentration indices and associated standard errors (s.e.) are estimated based on Appendix B, Equation 2. The model coefficient (Beta) and associated standard errors (se) are estimated based on Appendix B, Equation 3. The absolute contributions (abs.) to income related inequalities in AEAs are calculated using Appendix B, Equation (4) and the percentage contributions are calculated as a proportion of the concentration index for AEAs.

F. Sensitivity analysis 1 – Alternate emergency admission measures.

Table F1. Results: sample means, concentration indices (con. index), and percentage contributions to income inequality in all cause EAs, AEAs, chronic AEAs and urgent AEAs for 2012 and 2018.

**Admission**	**AEAs**		**Chronic AEAs**		**Urgent AEAs**		**All-cause EAs**	
**Year**	**2012**	**2018**	**2012**	**2018**	**2012**	**2018**	**2012**	**2018**
**Mean**	212.1	230.0	63.4	70.6	183.2	194.7	730.5	856.5
(s.d.)	(82.2)	(90.7)	(27.9)	(31.1)	(73.0)	(78.7)	(251.8)	(299.7)
**Con. index**	-0.120	-0.108	-0.145	-0.146	-0.124	-0.111	-0.109	-0.095
(s.e.)	(0.002)	(0.002)	(0.003)	(0.003)	(0.002)	(0.003)	(0.002)	(0.002)
**Contribution** (%)								
**Income**	**27%**	**26%**	**39%**	**39%**	**26%**	**24%**	**31%**	**31%**
**Age/sex**	**2%**	**-3%**	**2%**	**-1%**	**1%**	**-3%**	**2%**	**-3%**
**LTCs**	**20%**	**7%**	**19%**	**11%**	**20%**	**8%**	**18%**	**10%**
COPD	12%	7%	10%	8%	14%	9%	10%	4%
CHD	10%	-4%	9%	1%	9%	-4%	9%	2%
Depression	0%	3%	0%	3%	0%	3%	0%	4%
Asthma	-1%	1%	1%	1%	-2%	1%	0%	2%
Atrial Fib.	-1%	-1%	-1%	-2%	-1%	-1%	-1%	-2%
**SDOH**	**39%**	**51%**	**37%**	**42%**	**40%**	**52%**	**35%**	**44%**
Ethnicity	0%	0%	0%	0%	0%	0%	0%	0%
Education	10%	14%	10%	12%	10%	14%	10%	14%
Social Class	5%	15%	11%	16%	5%	14%	6%	16%
Unemployment	13%	12%	11%	9%	14%	13%	11%	7%
Lives Alone	8%	3%	6%	2%	8%	4%	7%	3%
Urban	2%	6%	-1%	4%	3%	6%	1%	4%
**Region**	**12%**	**19%**	**4%**	**8%**	**12%**	**20%**	**14%**	**18%**
South East	3%	-4%	5%	0%	2%	-5%	1%	-7%
North West	6%	7%	3%	5%	6%	7%	7%	9%
London	1%	5%	0%	1%	1%	6%	1%	3%
West Midl.	1%	3%	-1%	0%	1%	4%	2%	3%
North East	2%	3%	0%	2%	2%	3%	3%	4%
East Midl.	-1%	1%	-2%	0%	0%	1%	0%	1%
Yorkshire	1%	2%	0%	0%	1%	2%	1%	3%
South West	-1%	1%	-1%	0%	-1%	1%	-1%	1%
**Total**	**100%**	**100%**	**100%**	**100%**	**100%**	**100%**	**100%**	**100%**

The concentration indices and associated standard errors (s.e.) are estimated based on Appendix, Equation 2. All concentration indices are significant at the 99% confidence level respectively. The percentage contributions are calculated as a proportion of the concentration index for AEAs based on Appendix B, Equation 4. The contribution of the LTCs (COPD, CHD, Depression, Asthma, Atrial Fib.), SDOH factors (Ethnicity, Education, Social Class, Unemployment, Lives Alone, Urban), and regions (East of England as reference) are reported individually and as aggregates in bold.

Table F2. Expanded results for all-cause Chronic AEAs in 2012

Variable	Mean	s.d.	Beta	s.e.	Con. index	s.e.	Cont.	%
Logged Income	10.52	0.25	-26.99	2.73	0.014	0.000	-0.061	39%
Male 0 to 17	861.46	264.34	0.02	0.00	-0.012	0.002	-0.003	2%
Male 18 to 34	905.04	511.09	-0.01	0.00	-0.035	0.004	0.003	-2%
Male 35 to 49	816.84	211.10	0.01	0.00	0.028	0.002	0.003	-2%
Male 50 to 64	697.04	172.54	0.00	0.01	0.018	0.002	0.000	0%
Male 65 to 79	457.57	168.94	-0.02	0.01	0.012	0.002	-0.002	1%
Male 80 plus	139.11	61.86	-0.02	0.01	0.016	0.003	-0.001	1%
Female 0 to 17	820.41	252.83	0.01	0.00	-0.013	0.002	-0.001	1%
Female 18 to 34	896.58	473.44	0.01	0.00	-0.039	0.004	-0.005	3%
Female 35 to 49	830.34	201.18	0.00	0.00	0.035	0.002	-0.001	1%
Female 50 to 64	714.86	183.19	0.01	0.01	0.022	0.002	0.003	-2%
Female 65 to 79	506.48	183.13	0.04	0.01	0.008	0.002	0.003	-2%
Female 80 plus	230.10	105.20	0.05	0.01	-0.010	0.003	-0.002	1%
COPD	1.65	0.61	4.75	0.74	-0.130	0.002	-0.016	10%
CHD	3.46	0.98	3.74	0.65	-0.072	0.002	-0.015	9%
Depression	5.84	1.86	-0.02	0.14	-0.041	0.002	0.000	0%
Asthma	5.98	0.85	0.55	0.38	-0.025	0.001	-0.001	1%
Atrial Fibrillation	1.47	0.48	-9.16	1.09	-0.011	0.002	0.002	-1%
White (British)	Ref							
White (other)	0.00	0.07	-4.44	2.30	0.390	0.106	0.000	0%
Black	0.01	0.11	-5.60	2.79	-0.180	0.046	0.000	0%
Asian	0.04	0.21	-3.74	1.69	-0.152	0.037	0.000	0%
Mixed/Other	0.00	0.02	-7.46	8.95	-0.630	0.394	0.000	0%
Education Level 4	Ref							
Education Level 3	0.03	0.17	-0.70	1.83	-0.351	0.042	0.000	0%
Education Level 1/2	0.45	0.50	-0.06	0.66	-0.211	0.013	0.000	0%
Education None	0.14	0.35	8.13	1.20	-0.853	0.020	-0.016	10%
NSSEC3 Level 1	Ref							
NSSEC3 Level 2	0.01	0.10	-3.08	1.83	0.165	0.054	0.000	0%
NSSEC3 Level 3	0.55	0.50	2.28	0.71	-0.851	0.006	-0.017	11%
NSSEC3 Level 4	0.00	0.03	8.18	6.30	-0.855	0.349	0.000	0%
Unemployment	0.06	0.03	84.64	19.45	-0.209	0.003	-0.018	11%
Living Alone	0.30	0.06	36.98	6.44	-0.053	0.002	-0.009	6%
Urban	0.82	0.38	-0.51	0.66	-0.273	0.016	0.002	-1%
EoE	Ref							
SE	0.16	0.37	-7.07	0.76	0.440	0.018	-0.008	5%
NW	0.14	0.34	5.39	1.06	-0.403	0.020	-0.005	3%
Ldn	0.14	0.35	-0.11	1.00	0.521	0.019	0.000	0%
WM	0.11	0.31	-2.80	0.95	-0.366	0.021	0.002	-1%
NE	0.05	0.22	0.21	1.50	-0.524	0.036	0.000	0%
EM	0.08	0.28	-7.58	1.01	-0.317	0.023	0.003	-2%
Yrks	0.10	0.30	-0.17	0.93	-0.234	0.024	0.000	0%
SW	0.10	0.30	-10.40	1.09	-0.128	0.019	0.002	-1%
Constant			261.80	31.38				

The concentration indices and associated standard errors (s.e.) are estimated based on Appendix B, Equation 2. The model coefficient (Beta) and associated standard errors (se) are estimated based on Appendix B, Equation 3. The absolute contributions (abs.) to income related inequalities in AEAs are calculated using Appendix B, Equation (4) and the percentage contributions are calculated as a proportion of the concentration index for AEAs.

Table F3. Expanded results for Chronic AEAs in 2018

Variable	Mean	s.d.	Beta	s.e.	Con. index	s.e.	Cont.	%
Logged Income	10.67	0.22	-38.07	3.40	0.012	0.000	-0.067	39%
Male 0 to 17	902.13	303.22	0.01	0.00	-0.004	0.002	0.000	0%
Male 18 to 34	926.64	608.08	0.00	0.00	-0.027	0.005	0.001	-1%
Male 35 to 49	796.95	265.19	0.01	0.00	0.046	0.002	0.003	-2%
Male 50 to 64	763.95	181.06	0.02	0.01	0.018	0.002	0.005	-3%
Male 65 to 79	520.20	193.67	-0.01	0.01	0.005	0.002	0.000	0%
Male 80 plus	164.34	72.72	-0.01	0.01	0.025	0.003	-0.001	0%
Female 0 to 17	858.23	289.51	0.02	0.00	-0.004	0.002	-0.001	1%
Female 18 to 34	899.72	547.22	0.01	0.00	-0.031	0.005	-0.002	1%
Female 35 to 49	809.20	232.84	-0.01	0.01	0.046	0.002	-0.007	4%
Female 50 to 64	787.10	190.10	0.01	0.01	0.018	0.002	0.002	-1%
Female 65 to 79	571.03	209.73	0.02	0.01	0.009	0.002	0.001	-1%
Female 80 plus	243.36	108.31	0.07	0.01	0.004	0.003	0.001	-1%
COPD	1.93	0.71	3.51	0.82	-0.140	0.002	-0.013	8%
CHD	3.24	0.94	0.35	0.79	-0.080	0.002	-0.001	1%
Depression	9.24	2.41	0.70	0.14	-0.058	0.002	-0.005	3%
Asthma	6.02	0.93	0.72	0.46	-0.033	0.001	-0.002	1%
Atrial Fibrillation	1.90	0.66	-4.61	0.99	-0.021	0.002	0.003	-2%
White (British)	Ref							
White (other)	0.00	0.07	-2.53	2.89	0.507	0.108	0.000	0%
Black	0.01	0.11	-9.50	3.02	0.063	0.044	0.000	0%
Asian	0.04	0.21	3.61	1.92	-0.068	0.038	0.000	0%
Mixed/Other	0.00	0.02	-74.19	18.72	-0.414	0.334	0.000	0%
Education Level 4	Ref							
Education Level 3	0.03	0.17	0.73	1.84	-0.356	0.042	0.000	0%
Education Level 1/2	0.45	0.50	0.94	0.75	-0.226	0.014	-0.001	1%
Education None	0.14	0.35	10.43	1.39	-0.878	0.021	-0.019	11%
NSSEC3 Level 1	Ref							
NSSEC3 Level 2	0.01	0.10	0.37	1.85	0.098	0.062	0.000	0%
NSSEC3 Level 3	0.55	0.50	4.22	0.83	-0.832	0.006	-0.027	16%
NSSEC3 Level 4	0.00	0.03	8.96	6.10	-0.778	0.318	0.000	0%
Unemployment	0.06	0.03	82.57	21.61	-0.196	0.003	-0.015	9%
Living Alone	0.30	0.06	17.30	6.96	-0.048	0.002	-0.003	2%
Urban	0.82	0.38	3.47	0.73	-0.167	0.016	-0.007	4%
EoE	Ref							
SE	0.16	0.37	-0.57	1.06	0.448	0.019	-0.001	0%
NW	0.14	0.34	11.60	1.22	-0.369	0.019	-0.008	5%
Ldn	0.14	0.35	-1.32	1.42	0.650	0.019	-0.002	1%
WM	0.11	0.31	0.20	1.23	-0.292	0.021	0.000	0%
NE	0.05	0.22	6.63	1.71	-0.582	0.037	-0.003	2%
EM	0.08	0.28	1.21	1.18	-0.192	0.025	0.000	0%
Yrks	0.10	0.30	1.00	1.30	-0.358	0.022	-0.001	0%
SW	0.10	0.30	-3.53	1.11	-0.130	0.018	0.001	0%
Constant			381.21	38.57				

The concentration indices and associated standard errors (s.e.) are estimated based on Appendix B, Equation 2. The model coefficient (Beta) and associated standard errors (se) are estimated based on Appendix B, Equation 3. The absolute contributions (abs.) to income related inequalities in AEAs are calculated using Appendix B, Equation (4) and the percentage contributions are calculated as a proportion of the concentration index for AEAs.

Table F4. Expanded results for Urgent AEAs in 2012

Variable	Mean	s.d.	Beta	s.e.	Con. index	s.e.	Cont.	%
Logged Income	10.52	0.25	-44.90	7.10	0.014	0.000	-0.035	26%
Male 0 to 17	861.46	264.34	0.03	0.01	-0.012	0.002	-0.002	1%
Male 18 to 34	905.04	511.09	0.00	0.00	-0.035	0.004	0.001	0%
Male 35 to 49	816.84	211.10	0.05	0.01	0.028	0.002	0.006	-4%
Male 50 to 64	697.04	172.54	0.02	0.02	0.018	0.002	0.001	-1%
Male 65 to 79	457.57	168.94	-0.07	0.02	0.012	0.002	-0.002	2%
Male 80 plus	139.11	61.86	-0.04	0.03	0.016	0.003	0.000	0%
Female 0 to 17	820.41	252.83	0.02	0.01	-0.013	0.002	-0.001	1%
Female 18 to 34	896.58	473.44	0.02	0.00	-0.039	0.004	-0.003	2%
Female 35 to 49	830.34	201.18	0.00	0.01	0.035	0.002	-0.001	1%
Female 50 to 64	714.86	183.19	0.02	0.02	0.022	0.002	0.001	-1%
Female 65 to 79	506.48	183.13	0.07	0.02	0.008	0.002	0.001	-1%
Female 80 plus	230.10	105.20	0.24	0.02	-0.010	0.003	-0.003	2%
COPD	1.65	0.61	15.76	1.86	-0.130	0.002	-0.019	14%
CHD	3.46	0.98	9.35	1.62	-0.072	0.002	-0.013	9%
Depression	5.84	1.86	0.05	0.34	-0.041	0.002	0.000	0%
Asthma	5.98	0.85	-2.95	0.97	-0.025	0.001	0.002	-2%
Atrial Fibrillation	1.47	0.48	-17.20	2.74	-0.011	0.002	0.001	-1%
White (British)	Ref							
White (other)	0.00	0.07	-22.64	6.70	0.390	0.106	0.000	0%
Black	0.01	0.11	-26.64	6.93	-0.180	0.046	0.000	0%
Asian	0.04	0.21	-4.55	4.46	-0.152	0.037	0.000	0%
Mixed/Other	0.00	0.02	17.91	34.51	-0.630	0.394	0.000	0%
Education Level 4	Ref							
Education Level 3	0.03	0.17	13.33	5.66	-0.351	0.042	-0.001	1%
Education Level 1/2	0.45	0.50	1.99	1.78	-0.211	0.013	-0.001	1%
Education None	0.14	0.35	17.04	3.02	-0.853	0.020	-0.011	8%
NSSEC3 Level 1	Ref							
NSSEC3 Level 2	0.01	0.10	-4.11	4.73	0.165	0.054	0.000	0%
NSSEC3 Level 3	0.55	0.50	2.76	1.92	-0.851	0.006	-0.007	5%
NSSEC3 Level 4	0.00	0.03	22.66	13.98	-0.855	0.349	0.000	0%
Unemployment	0.06	0.03	267.98	49.31	-0.209	0.003	-0.019	14%
Living Alone	0.30	0.06	128.90	16.94	-0.053	0.002	-0.011	8%
Urban	0.82	0.38	3.24	1.70	-0.273	0.016	-0.004	3%
EoE	Ref							
SE	0.16	0.37	-8.13	2.02	0.440	0.018	-0.003	2%
NW	0.14	0.34	26.21	2.66	-0.403	0.020	-0.008	6%
Ldn	0.14	0.35	-3.04	2.61	0.521	0.019	-0.001	1%
WM	0.11	0.31	4.44	2.41	-0.366	0.021	-0.001	1%
NE	0.05	0.22	23.28	3.61	-0.524	0.036	-0.003	3%
EM	0.08	0.28	-4.08	2.54	-0.317	0.023	0.001	0%
Yrks	0.10	0.30	7.15	2.43	-0.234	0.024	-0.001	1%
SW	0.10	0.30	-11.05	2.85	-0.128	0.019	0.001	-1%
Constant			400.74	81.44				

The concentration indices and associated standard errors (s.e.) are estimated based on Appendix B, Equation 2. The model coefficient (Beta) and associated standard errors (se) are estimated based on Appendix B, Equation 3. The absolute contributions (abs.) to income related inequalities in AEAs are calculated using Appendix B, Equation (4) and the percentage contributions are calculated as a proportion of the concentration index for AEAs.

Table F5. Expanded results for Urgent AEAs in 2018

Variable	Mean	s.d.	Beta	s.e.	Con. index	s.e.	Cont.	%
Logged Income	10.67	0.22	-49.18	8.98	0.012	0.000	-0.031	24%
Male 0 to 17	902.13	303.22	0.03	0.01	-0.004	0.002	-0.001	1%
Male 18 to 34	926.64	608.08	0.00	0.00	-0.027	0.005	0.000	0%
Male 35 to 49	796.95	265.19	0.02	0.01	0.046	0.002	0.003	-3%
Male 50 to 64	763.95	181.06	0.05	0.02	0.018	0.002	0.003	-3%
Male 65 to 79	520.20	193.67	-0.05	0.02	0.005	0.002	-0.001	1%
Male 80 plus	164.34	72.72	0.00	0.03	0.025	0.003	0.000	0%
Female 0 to 17	858.23	289.51	0.04	0.01	-0.004	0.002	-0.001	1%
Female 18 to 34	899.72	547.22	0.01	0.00	-0.031	0.005	-0.002	1%
Female 35 to 49	809.20	232.84	-0.02	0.01	0.046	0.002	-0.004	3%
Female 50 to 64	787.10	190.10	0.03	0.02	0.018	0.002	0.002	-2%
Female 65 to 79	571.03	209.73	0.02	0.02	0.009	0.002	0.001	-1%
Female 80 plus	243.36	108.31	0.28	0.02	0.004	0.003	0.001	-1%
COPD	1.93	0.71	8.26	2.11	-0.140	0.002	-0.011	9%
CHD	3.24	0.94	-4.43	2.01	-0.080	0.002	0.006	-5%
Depression	9.24	2.41	1.64	0.35	-0.058	0.002	-0.005	3%
Asthma	6.02	0.93	1.15	1.21	-0.033	0.001	-0.001	1%
Atrial Fibrillation	1.90	0.66	-4.42	2.55	-0.021	0.002	0.001	-1%
White (British)	Ref							
White (other)	0.00	0.07	-13.29	6.75	0.507	0.108	0.000	0%
Black	0.01	0.11	-36.51	6.86	0.063	0.044	0.000	0%
Asian	0.04	0.21	22.69	5.71	-0.068	0.038	0.000	0%
Mixed/Other	0.00	0.02	-167.22	47.26	-0.414	0.334	0.000	0%
Education Level 4	Ref							
Education Level 3	0.03	0.17	12.75	5.27	-0.356	0.042	-0.001	1%
Education Level 1/2	0.45	0.50	6.87	2.00	-0.226	0.014	-0.004	3%
Education None	0.14	0.35	22.69	3.65	-0.878	0.021	-0.015	11%
NSSEC3 Level 1	Ref							
NSSEC3 Level 2	0.01	0.10	4.75	5.18	0.098	0.062	0.000	0%
NSSEC3 Level 3	0.55	0.50	7.87	2.19	-0.832	0.006	-0.018	14%
NSSEC3 Level 4	0.00	0.03	90.67	12.58	-0.778	0.318	0.000	0%
Unemployment	0.06	0.03	270.56	57.41	-0.196	0.003	-0.017	13%
Living Alone	0.30	0.06	77.85	18.82	-0.048	0.002	-0.006	4%
Urban	0.82	0.38	10.55	1.85	-0.167	0.016	-0.007	6%
EoE	Ref							
SE	0.16	0.37	17.99	2.66	0.448	0.019	0.007	-5%
NW	0.14	0.34	36.95	2.97	-0.369	0.019	-0.010	7%
Ldn	0.14	0.35	-15.43	3.72	0.650	0.019	-0.007	6%
WM	0.11	0.31	29.77	3.39	-0.292	0.021	-0.005	4%
NE	0.05	0.22	31.04	3.98	-0.582	0.037	-0.005	4%
EM	0.08	0.28	19.57	2.96	-0.192	0.025	-0.002	1%
Yrks	0.10	0.30	16.97	3.56	-0.358	0.022	-0.003	2%
SW	0.10	0.30	22.05	2.75	-0.130	0.018	-0.002	1%
Constant			440.06	102.04				

The concentration indices and associated standard errors (s.e.) are estimated based on Appendix B, Equation 2. The model coefficient (Beta) and associated standard errors (se) are estimated based on Appendix B, Equation 3. The absolute contributions (abs.) to income related inequalities in AEAs are calculated using Appendix B, Equation (4) and the percentage contributions are calculated as a proportion of the concentration index for AEAs.

Table F6. Expanded results for All-cause EAs in 2012

Variable	Mean	s.d.	Beta	s.e.	Con. index	s.e.	Cont.	%
Logged Income	10.52	0.25	-184.79	21.91	0.014	0.000	-0.036	31%
Male 0 to 17	861.46	264.34	0.16	0.03	-0.012	0.002	-0.002	2%
Male 18 to 34	905.04	511.09	0.00	0.02	-0.035	0.004	0.000	0%
Male 35 to 49	816.84	211.10	0.19	0.03	0.028	0.002	0.006	-5%
Male 50 to 64	697.04	172.54	0.02	0.05	0.018	0.002	0.000	0%
Male 65 to 79	457.57	168.94	-0.17	0.07	0.012	0.002	-0.001	1%
Male 80 plus	139.11	61.86	0.05	0.10	0.016	0.003	0.000	0%
Female 0 to 17	820.41	252.83	0.15	0.04	-0.013	0.002	-0.002	2%
Female 18 to 34	896.58	473.44	0.07	0.02	-0.039	0.004	-0.003	3%
Female 35 to 49	830.34	201.18	-0.04	0.04	0.035	0.002	-0.002	1%
Female 50 to 64	714.86	183.19	0.09	0.05	0.022	0.002	0.002	-2%
Female 65 to 79	506.48	183.13	0.25	0.06	0.008	0.002	0.001	-1%
Female 80 plus	230.10	105.20	0.56	0.06	-0.010	0.003	-0.002	1%
COPD	1.65	0.61	40.13	5.69	-0.130	0.002	-0.012	10%
CHD	3.46	0.98	32.57	5.07	-0.072	0.002	-0.011	9%
Depression	5.84	1.86	0.64	1.03	-0.041	0.002	0.000	0%
Asthma	5.98	0.85	-1.06	3.02	-0.025	0.001	0.000	0%
Atrial Fibrillation	1.47	0.48	-57.47	8.50	-0.011	0.002	0.001	-1%
White (British)	Ref							
White (other)	0.00	0.07	-39.37	18.72	0.390	0.106	0.000	0%
Black	0.01	0.11	-66.13	22.78	-0.180	0.046	0.000	0%
Asian	0.04	0.21	-23.76	14.83	-0.152	0.037	0.000	0%
Mixed/Other	0.00	0.02	38.52	106.18	-0.630	0.394	0.000	0%
Education Level 4	Ref							
Education Level 3	0.03	0.17	25.12	18.14	-0.351	0.042	0.000	0%
Education Level 1/2	0.45	0.50	7.28	5.53	-0.211	0.013	-0.001	1%
Education None	0.14	0.35	63.16	9.56	-0.853	0.020	-0.011	9%
NSSEC3 Level 1	Ref							
NSSEC3 Level 2	0.01	0.10	-11.78	15.27	0.165	0.054	0.000	0%
NSSEC3 Level 3	0.55	0.50	10.86	5.99	-0.851	0.006	-0.007	6%
NSSEC3 Level 4	0.00	0.03	60.36	31.70	-0.855	0.349	0.000	0%
Unemployment	0.06	0.03	705.07	155.47	-0.209	0.003	-0.013	11%
Living Alone	0.30	0.06	377.45	53.59	-0.053	0.002	-0.008	7%
Urban	0.82	0.38	5.22	5.34	-0.273	0.016	-0.002	1%
EoE	Ref							
SE	0.16	0.37	-11.62	5.97	0.440	0.018	-0.001	1%
NW	0.14	0.34	104.63	8.15	-0.403	0.020	-0.008	7%
Ldn	0.14	0.35	-13.45	8.11	0.521	0.019	-0.001	1%
WM	0.11	0.31	36.71	7.50	-0.366	0.021	-0.002	2%
NE	0.05	0.22	107.12	11.01	-0.524	0.036	-0.004	3%
EM	0.08	0.28	-12.14	7.64	-0.317	0.023	0.000	0%
Yrks	0.10	0.30	42.25	7.17	-0.234	0.024	-0.001	1%
SW	0.10	0.30	-32.64	9.17	-0.128	0.019	0.001	-1%
Constant			1683.98	251.71				

The concentration indices and associated standard errors (s.e.) are estimated based on Appendix B, Equation 2. The model coefficient (Beta) and associated standard errors (se) are estimated based on Appendix B, Equation 3. The absolute contributions (abs.) to income related inequalities in AEAs are calculated using Appendix B, Equation (4) and the percentage contributions are calculated as a proportion of the concentration index for AEAs.

Table F7. Expanded results for All-cause EAs in 2018

Variable	Mean	s.d.	Beta	s.e.	Con. index	s.e.	Cont.	%
Logged Income	10.67	0.22	-241.68	30.84	0.012	0.000	-0.035	31%
Male 0 to 17	902.13	303.22	0.16	0.04	-0.004	0.002	-0.001	1%
Male 18 to 34	926.64	608.08	-0.01	0.02	-0.027	0.005	0.000	0%
Male 35 to 49	796.95	265.19	0.11	0.04	0.046	0.002	0.005	-4%
Male 50 to 64	763.95	181.06	0.17	0.05	0.018	0.002	0.003	-2%
Male 65 to 79	520.20	193.67	-0.09	0.07	0.005	0.002	0.000	0%
Male 80 plus	164.34	72.72	0.07	0.11	0.025	0.003	0.000	0%
Female 0 to 17	858.23	289.51	0.23	0.05	-0.004	0.002	-0.001	1%
Female 18 to 34	899.72	547.22	0.05	0.02	-0.031	0.005	-0.002	2%
Female 35 to 49	809.20	232.84	-0.10	0.05	0.046	0.002	-0.004	4%
Female 50 to 64	787.10	190.10	0.13	0.06	0.018	0.002	0.002	-2%
Female 65 to 79	571.03	209.73	0.06	0.06	0.009	0.002	0.000	0%
Female 80 plus	243.36	108.31	0.84	0.06	0.004	0.003	0.001	-1%
COPD	1.93	0.71	12.98	7.24	-0.140	0.002	-0.004	4%
CHD	3.24	0.94	7.40	6.83	-0.080	0.002	-0.002	2%
Depression	9.24	2.41	6.35	1.17	-0.058	0.002	-0.004	4%
Asthma	6.02	0.93	11.63	4.17	-0.033	0.001	-0.003	2%
Atrial Fibrillation	1.90	0.66	-37.04	8.50	-0.021	0.002	0.002	-2%
White (British)	Ref							
White (other)	0.00	0.07	-30.16	26.75	0.507	0.108	0.000	0%
Black	0.01	0.11	-84.54	27.35	0.063	0.044	0.000	0%
Asian	0.04	0.21	59.18	20.39	-0.068	0.038	0.000	0%
Mixed/Other	0.00	0.02	-737.40	172.74	-0.414	0.334	0.000	0%
Education Level 4	Ref							
Education Level 3	0.03	0.17	29.45	19.16	-0.356	0.042	0.000	0%
Education Level 1/2	0.45	0.50	27.15	6.97	-0.226	0.014	-0.003	3%
Education None	0.14	0.35	83.52	12.58	-0.878	0.021	-0.012	11%
NSSEC3 Level 1	Ref							
NSSEC3 Level 2	0.01	0.10	40.61	18.95	0.098	0.062	0.000	0%
NSSEC3 Level 3	0.55	0.50	33.97	7.66	-0.832	0.006	-0.018	16%
NSSEC3 Level 4	0.00	0.03	181.22	49.34	-0.778	0.318	0.000	0%
Unemployment	0.06	0.03	555.96	194.65	-0.196	0.003	-0.008	7%
Living Alone	0.30	0.06	211.59	66.03	-0.048	0.002	-0.004	3%
Urban	0.82	0.38	25.41	6.43	-0.167	0.016	-0.004	4%
EoE	Ref							
SE	0.16	0.37	98.94	9.33	0.448	0.019	0.008	-8%
NW	0.14	0.34	176.03	10.21	-0.369	0.019	-0.010	9%
Ldn	0.14	0.35	-35.56	13.37	0.650	0.019	-0.004	4%
WM	0.11	0.31	95.41	11.46	-0.292	0.021	-0.004	3%
NE	0.05	0.22	140.82	13.57	-0.582	0.037	-0.005	4%
EM	0.08	0.28	60.60	9.98	-0.192	0.025	-0.001	1%
Yrks	0.10	0.30	83.56	12.80	-0.358	0.022	-0.004	3%
SW	0.10	0.30	79.25	9.75	-0.130	0.018	-0.001	1%
Constant			2261.09	349.81				

The concentration indices and associated standard errors (s.e.) are estimated based on Appendix B, Equation 2. The model coefficient (Beta) and associated standard errors (se) are estimated based on Appendix B, Equation 3. The absolute contributions (abs.) to income related inequalities in AEAs are calculated using Appendix B, Equation (4) and the percentage contributions are calculated as a proportion of the concentration index for AEAs.

G. Sensitivity analysis 2 – Using a negative binomial model to estimate the determinants of health care utilisation model.

Figure G1. The percentage contribution of each factor to income-inequality in AEAs in 2012/13 and 2018/19 based on the marginal effects from negative binomial models reported in Table G1

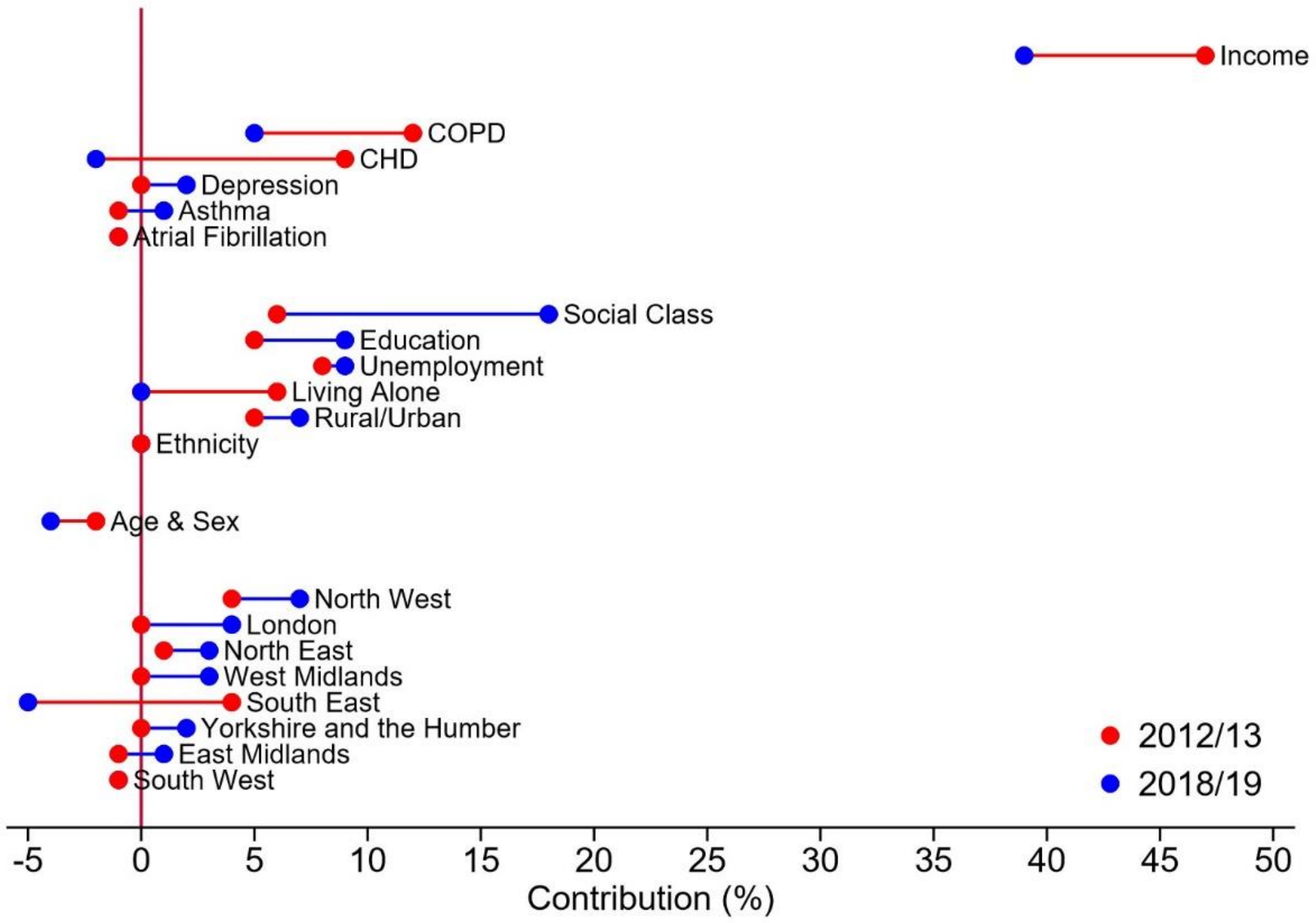

Table G1. Marginal effects from the determinants of health care use models where avoidable admissions is the outcome variable estimated as a negative binomial model with robust standard errors.

	2012		2018	
**Variable**	**Marginal effect**	**s.d.**	**Marginal effect**	**s.e.**
Logged Income	-91.80	8.49	-98.07	11.16
Male 0 to 17	0.04	0.01	0.03	0.01
Male 18 to 34	-0.01	0.01	0.00	0.01
Male 35 to 49	0.05	0.01	0.02	0.01
Male 50 to 64	-0.01	0.02	0.07	0.02
Male 65 to 79	-0.07	0.02	-0.05	0.02
Male 80 plus	0.01	0.04	0.01	0.03
Female 0 to 17	0.02	0.01	0.05	0.01
Female 18 to 34	0.02	0.01	0.01	0.01
Female 35 to 49	0.02	0.01	-0.01	0.02
Female 50 to 64	0.04	0.02	0.03	0.02
Female 65 to 79	0.08	0.02	0.02	0.02
Female 80 plus	0.25	0.02	0.33	0.02
COPD	14.90	1.96	5.98	2.26
CHD	10.34	1.75	-2.71	2.23
Depression	-0.28	0.36	1.43	0.39
Asthma	-2.68	1.07	1.95	1.38
Atrial Fibrillation	-19.72	3.08	-5.91	2.79
White (British)				
White (other)	-35.68	11.31	-36.22	13.04
Black	-25.93	8.41	-34.76	10.51
Asian	-2.17	5.14	25.82	6.34
Mixed/Other	-1.91	24.30	-190.57	81.78
Education Level 4				
Education Level 3	11.33	6.29	9.47	5.79
Education Level 1/2	0.19	2.18	5.65	2.39
Education None	9.59	3.26	16.28	3.86
NSSEC3 Level 1				
NSSEC3 Level 2	-14.85	7.17	3.54	7.24
NSSEC3 Level 3	3.28	2.32	12.39	2.64
NSSEC3 Level 4	39.23	11.21	60.87	11.76
Unemployment	175.28	48.20	224.89	60.48
Living Alone	98.67	19.84	0.63	23.68
Urban	5.70	2.01	14.65	2.21
EoE				
SE	-16.43	2.42	21.83	3.18
NW	18.86	2.89	42.37	3.33
Ldn	-0.09	3.02	-13.04	4.65
WM	1.31	2.75	31.17	3.75
NE	12.05	3.60	30.56	4.10
EM	-12.94	2.91	21.26	3.35
Y&H	4.77	2.68	15.07	3.74
SW	-22.27	3.33	18.86	3.21
